# Hybrid materials for wastewater treatment: synergistic coupling of *Neochloris oleoabundans* and TiO_2_ nanoparticles†

**DOI:** 10.1039/d5na00236b

**Published:** 2025-05-12

**Authors:** Ilaria Zanoni, Sara Amadori, Andrea Brigliadori, Anna Luisa Costa, Simona Ortelli, Pierluigi Giacò, Costanza Baldisserotto, Simonetta Pancaldi, Magda Blosi

**Affiliations:** a CNR-ISSMC, National Research Council of Italy, Institute of Science, Technology and Sustainability for Ceramics Faenza Italy magda.blosi@issmc.cnr.it; b Department of Chemical Science, Life and Environmental Sustainability, Parma University Parma Italy; c Department of Environmental and Prevention Sciences, University of Ferrara 44121 Ferrara Italy

## Abstract

In this work, we combined microalgae's sorptive properties with titania-based nanoparticles' photocatalytic capabilities to develop technologies applicable to wastewater treatment while also providing valuable insights into the innovation of adsorption technologies. The coupling of *Neochloris oleoabundans* biomass with an inorganic nanophase enables the formation of hybrid materials integrating heavy metal adsorption with photocatalytic action. To prepare the samples, we employed a water-based colloidal method followed by a spray freeze granulation treatment. The preparation process was followed by comprehensive physicochemical characterization from the wet precursors to the final hybrid granules. Key performance indicators, including adsorption and photocatalytic activity, were assessed using two model contaminants: copper ions (for heavy metal adsorption) and Rhodamine B (for photocatalysis). The results revealed a synergistic effect of the hybrid nanomaterials, significantly enhancing the Cu^2+^ adsorption capacity of the biomass, which increases from 30 mg g^−1^ to 250 mg g^−1^ when coupled with the inorganic phase and is likely due to the supporting and dispersing role of the inorganic nanoparticles on the biomass. The adsorption experimental values followed the Freundlich isothermal model and pseudo-second-order kinetic model, indicating that the adsorption occurred primarily through a multimolecular layer adsorption process, consistent with chemisorption mechanisms. The photocatalytic performance of the inorganic counterpart was preserved when coupled with the microalgae, with TiO_2_–SiO_2_/biomass achieving complete Rhodamine B degradation within 1 hour.

## Abstract 2

*Neochloris oleoabundans* microalgae coupled with TiO_2_ NPs provides a multifunctional hybrid material for water treatment application with enhanced biomass adsorption capacity and preserved photocatalytic activity.

## Graphical abstract

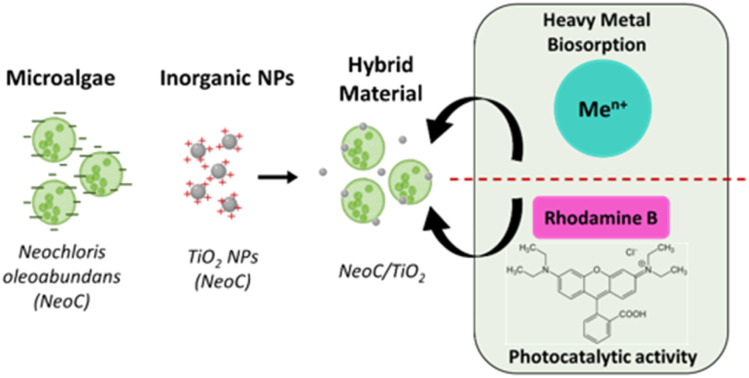

## 1. Introduction

Bioremediation has emerged as a promising technology for treating industrial effluents in wastewater remediation and advanced purification.

Among the various materials studied for this purpose, algae and microalgae have gained significant attention.^1–3^ These microorganisms can transform wastewater, CO_2_, and organic residues into valuable biomass for applications like biofuels, effectively turning waste into a resource.^4–6^ Biosorption by microalgae is a competitive and cost-efficient alternative to conventional methods.^4,5,7^ It has been exploited based on a favorable combination of abundance in seawater and freshwater, reusability, and high metal sorption capacities, offering possibilities for metal recovery and biosorbent regeneration afterward. Heavy metals, even in trace amounts, are significant environmental pollutants that pose serious risks to aquatic life and human health. Long-term exposure can lead to bioaccumulation, causing poisoning and disrupting ecosystems. At present, living and dead microalgae are being explored in laboratory settings, showing the ability to remove heavy metals and dyes^8,9^ and making biosorption a promising approach to remediate aqueous solutions.

Research suggests that nonliving cells may be more advantageous than living ones in terms of processing and improved applicability; in fact, they do not need nutrients and are much less affected by the physicochemical properties of the super-natant solutions containing metals. The cell walls of macro/microalgae, composed mainly of polysaccharides, proteins, and lipids, carry functional groups that give the surface a negative charge and a high affinity for binding metal cations through electrostatic interactions. Metal biosorption by non-living microalgae involves several mechanisms, including electrostatic interactions, van der Waals forces, covalent bonding, redox reactions, and biomineralization processes.^10^ Despite the notable potential of microalgae, new approaches and designs are necessary to enhance their advantages over conventional methods and boost their application spectra.

Recently, several methods have been introduced to improve the efficiency of water treatment, and a key area has been the development of innovative hybrid materials that combine multiple techniques, including photocatalysis, adsorption, and enzyme-driven reactions.^11^

In this perspective, combining biosorbent microalgae with photocatalytic inorganic nanoparticles (NPs), as like as TiO_2_, represents an unexplored area for developing new hybrid multifunctional materials. TiO_2_ nanoparticles are widely used in water treatment due to their photocatalytic properties, which enable the degradation of pollutants under UV light. Their high surface area and stability make them an efficient and durable option for water purification processes.

We present a multifunctional hybrid material that combines adsorption and photocatalytic properties, enhanced by *Neochloris oleoabundans* and TiO_2_ NPs, respectively.

In our previous work, we showed advantageous synergistic effects by coupling *Chlorella vulgaris* biomass with TiO_2_ nanoparticles (NPs), and here, we report the experiments carried out on TiO_2_ nanoparticles (NPs) combined with *Neochloris oleoabundans*, confirming a similar synergistic effect improving adsorption. This innovative approach could pave the way to a novel frontier in the design of hybrid nanomaterials and open new horizons for algae-based bioremediation and broader wastewater treatment applications. Most studies dealing with inorganic nanoparticles interacting with microalgae have focused on the ecotoxicological aspects of the nanophases.^12–14^

## 2. Materials and methods

Titanium dioxide nanopowder (Aeroxide® P25, Evonik) and silica nanosol (Ludox HS-40, Grace Davison) are the primary components for constructing the inorganic porous matrix. The organic counterpart, *Neochloris oleoabundans* (NeoC), microalgae, is incorporated into the inorganic structure. The microalgae, sourced from the University of Ferrara (UNIFE), were freshly cultivated in a solution containing 0.27 g per L NaCl and extracted from the bioreactor at a concentration of 0.22 g L^−1^, where they were designated as NeoC.

### 2.1. *Neochloris oleoabundans* cultivation

*Neochloris oleoabundans* UTEX 1185 from the Culture Collection of Algae at the University of Texas at Austin (USA; https://www.utex.org) was inoculated in a 100 L capacity coaxial photobioreactor filled with BG11 medium. The culture was illuminated with 60 μmol photons m^−2^ s^−1^ of photosynthetically active radiation with a 16 : 8 h light/dark photoperiod and insufflated with sterile air with 0.5 : 3.5 h bubbling/static cycles.^15^ The algae samples were collected after 28 days when the algae reached the stationary growth phase. The dry weight of the algal culture was evaluated using pre-weighed and pre-dried glass fiber filters (Whatman GF/C; 1.2 μm pore size); samples were filtered, rinsed with distilled water, dried for 72 h at 60 °C and then weighted.^15^ Cultivation modes affect the morphology, biochemical composition, and antioxidant and anti-inflammatory properties of the green microalga *Neochloris oleoabundans*.^16^ The final dry weight of the culture was 0.22 g L^−1^.

### 2.2 Materials preparation at the colloidal stage

TiO_2_ or TiO_2_–SiO_2_ inorganic nanoparticles (NPs) and NeoC microalgae were first heterocoagulated in bi-distilled water suspensions and then granulated. Heterocoagulation is the colloidal process that promotes interactions between biomass and inorganic nanoparticles through a self-assembly mechanism driven by electrostatic surface force. We prepared the NeoC/TiO_2_ sample by adding TiO_2_ nanopowder to 110 mL of NeoC suspension (0.22 g L^−1^) to achieve a final concentration of 1.5 wt% for TiO_2_ nanoparticles and a NeoC/TiO_2_ weight ratio of 1.4%. This mixture was then stirred gently for 24 hours.

For the preparation of the TiO_2_–SiO_2_ sample, a TiO_2_ nanosuspension (0.75 wt%) was mixed with a SiO_2_ nanosuspension (Ludox HS-40, 2.25 wt%) before the addition of microalgae. Silica was introduced into the TiO_2_ nanosuspension according to the method previously developed by our research group^14,17^ and in a weight ratio TiO_2_ : SiO_2_ 1 : 3, identified as the optimal composition maximizing the photocatalytic activity. Before adding the silica suspension (Ludox HS-40), the pH was adjusted from pH 9.7 to 4 by using a cation exchange resin (Dowex 50 WX8 20–50, LennTech).

To prepare the three-component NeoC/TiO_2_–SiO_2_ sample, the TiO_2_ and SiO_2_ suspensions were mixed first, followed by the addition of microalgae. The silica sol (2.25 wt%, pH 4) was slowly added to the TiO_2_ suspension (0.75 wt%) under stirring and treated by ball-milling for 24 hours to enhance the oxide interaction. Finally, the appropriate amount of NeoC suspension (0.22 g L^−1^) was added under gentle stirring for 1 hour to achieve a NeoC/TiO_2_–SiO_2_ weight ratio of 1.4%.

### 2.3 Colloidal characterization

The hydrodynamic diameters distribution and Zeta Potential (ZP) of microalgae suspensions were evaluated using a Zetasizer instrument (Zetasizer Nano-ZS, Malvern Instruments, Worcestershire, UK), based respectively on the dynamic light scattering (DLS) technique and the electrophoretic light scattering (ELS) technique. The size distribution (nm) is reported as intensity-weighted mean diameter derived from the cumulant analysis (*Z*-average) and is the average of three independent measurements. The Zeta Potential (ZP) value is derived by the electrophoretic mobility using Smoluchowski's formula and represents the average of three independent measurements. The Zetasizer instrument is equipped with an automatic titrator (Malvern MPT-2 Multipurpose Titrator, Malvern Instrument, UK) used to perform titrations of zeta potential as a function of pH. A data point of interest that is usually determined during the titration run is the isoelectric point (IEP, IsoElectric Point), which represents the pH at which the electrical charges of the particles are neutralized (ZP value of 0 mV) and implies the maximum colloidal destabilization. Titration requires 10 mL of sample at 100 mg L^−1^ and uses 0.1 M HCl acid. The pH is constantly monitored with the in-line pH meter (MV 114-SC, Malvern Instrument). The Zetasizer instrument was also used to evaluate the NeoC coverage of TiO_2_ NPs, assessing the change in ZP values by titration. TiO_2_ NPs suspension was prepared at 100 mg L^−1^, and a 0.22 g per L suspension of NeoC was added by step of 50 μL till reaching a plateau in the ZP values.

### 2.4. Spray freeze granulation process

We used a spray-freeze drying method to convert nanosuspensions into micrometric powders with a lab-scale apparatus (Labscale Granulator LS-2, Powder Pro). A peristaltic pump atomized the suspension, and nitrogen gas at 0.4 bar passed through a 100 μm nozzle, spraying the suspension into a stirred liquid nitrogen bath, where each droplet froze instantly. The frozen droplets were treated by a freeze-drying system (LYO GT 2, SRK System Technik) under a pressure of 1.5 × 10^−1^ mbar and a temperature of −1 °C. Over 72 hours, sublimation occurred, producing a porous granulated powder. We labelled the resulting spray freeze dried samples with the code SFD.

### 2.5. Morphological characterization

The suspension of NeoC was dropped on a glass slide and observed with a 3D digital optical microscope (Hirox RH-2000) equipped with a high-intensity LED lamp (5700 K) and a magnification range of 35–5000×. The morphology of the SFD granules was analysed by using a Field Emission Scanning Electron Microscope, FE-SEM (Carl Zeiss Sigma NTS, Germany) and the particle size distribution was calculated on more than 120 units by using the ImageJ software.

### 2.6. Fourier transform infrared spectroscopy

FTIR analysis was performed on NeoC and NeoC-based granules. The measurements were obtained using a Nicolet iS5 spectrometer (Thermo Fisher Scientific Inc. – Waltham, MA, USA). Samples were mixed with KBr (1 : 100 ratio of granules: KBr) and analyzed in a wave number range between 400 and 4000 cm^−1^. The positions of the peaks were identified using the OMNIC software, and values were compared with the data in the literature.

### 2.7. Specific surface area by BET method

Specific surface areas of SFD samples were assessed by N_2_ physisorption technique (Sorpty 1750 CE instruments) throughout a single point analysis method (Brunauer–Emmett–Teller, BET). Samples were pre-treated under vacuum at 100 °C for 2 hours.

### 2.8. Adsorption tests

First, the elemental composition and trace elements intrinsically present in NeoC were evaluated through acid treatment of the dried suspension (previously treated at 80 °C overnight). 7 mg of NeoC were acid digested by adding 1 mL nitric acid (HNO_3_ 65%) and 1 mL of hydroxy peroxide (H_2_O_2_ 30%), then diluting with water at 10 mL. A set of different trace elements were quantified: Ca, Fe, K, Mg, Mn, Na, and Zn.

We evaluated the adsorption capacity of the SFD samples by testing the samples in presence of Cu^2+^ ions. We dispersed the SFD samples in a solution of CuCl_2_ (10 mg L^−1^) by keeping the system under stirring at a constant temperature of 25 °C for 30 min and at a working pH of 6. Adsorption tests were performed with 2.5 g L^−1^ of SFD granulated samples or 0.118 g L^−1^ of NeoC.^14,18^

To quantify the adsorption of Cu^2+^ we separated the SFD powder by centrifuging the suspensions at 4500 rpm for 40 min by using centrifugal filter units (polyethersulfone, Amicon filter 10 kDa). We quantified the non-absorbed Cu^2+^ ions by analysing the liquid phase using ICP-OES. The Cu^2+^ adsorption was tested against the Langmuir (eqn (1)) and Freundlich (eqn (2)) isotherm models:1
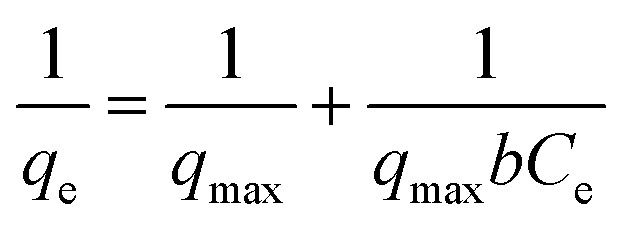
2
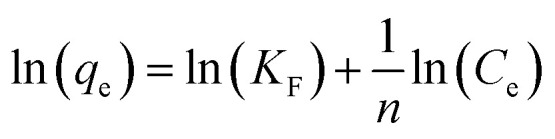
where for the Langmuir model *C*_e_ is the Cu^2+^ concentration at the equilibrium (mg L^−1^), *q*_max_ is the maximum adsorption capacity (mg g^−1^), and *b* is the Langmuir constant (L mg^−1^). For the Freundlich model, *C*_e_ is the Cu^2+^ concentration at the equilibrium (mg L^−1^), *q*_e_ is the amount of adsorbed ions at the equilibrium (mg g^−1^), *K*_F_ is the adsorption constant corresponding to adsorption capacity, whereas 1/*n* is the adsorption intensity. *n* depends on the adsorbate and adsorbent. Any value of *n*, higher than unity, favors the adsorption process.

Tests were carried out on volumes of 10 mL at increasing Cu^2+^ concentrations (1, 10, 100, 500 mg L^−1^) exposed to 1.18 mg of NeoC or 25 mg of granulated hybrid bio-sorbent.

The adsorption kinetics were followed using a pseudo-first (PFO), eqn (3), and pseudo-second-order (PSO), eqn (4):

3 ln(*q*_e_ − *q*_*t*_) = ln(*q*_e_) − *k*_1_*t*

4
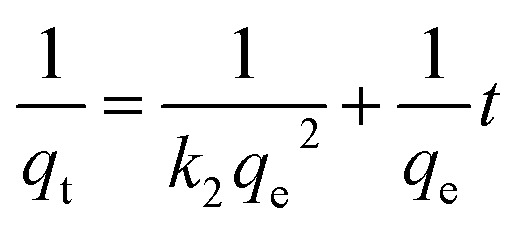


Kinetic evaluation tests were carried out on a volume of 200 mL at a concentration of 10 mg L^−1^ for CuCl_2_ and exposed to 11.79 mg NeoC (0.059 g L^−1^) or 500 mg of granulated hybrid bio-sorbent. Cu^2+^ biosorption kinetic was quantified by ICP-OES measurements on the treated solutions at increasing times (1, 5, 10, 15, 20, 30, 40, 50 min). To quantify the adsorption, after keeping the samples in contact with Cu^2+^, we centrifuged 10 mL of solution at 4500 rpm for 40 min by filtering the sample with centrifugal filter units (Polyethersulfone, Amicon filter 10 kDa). This way, we separated the adsorbent powders from the solution, and we quantified the non-absorbed ions employing ICP-OES assessment. The elemental composition of the microalgae, the sodium content of the washing water, and the adsorption performances of Cu^2+^ concentration in water were evaluated by inductively coupled plasma optical emission spectrometry using an ICP-OES 5100 – vertical dual view apparatus (Agilent Technologies, Santa Clara, CA, USA) coupled with OneNeb nebulizer and equipped with an Autosampler. The measurements were calibrated by the use of calibration curves with a correlation coefficient limit higher than 0.999. The calibration fit was linear, including a blank in calibration. The precision of the measurements expressed as relative standard deviation (RSD%) for the analysis was always less than 5%. The limit of detection (LOD) at the operative wavelength was 0.01 mg L^−1^ for all analysed elements. Samples were acid digested, adding a 10% volume of nitric acid (HNO_3_ 65%) and 10% of hydroxy peroxide (H_2_O_2_ 30%). Calibration curves were obtained in the range of 0.01–100 mg L^−1^, applying to the standards the same digestive procedure of samples.

### 2.9. Photocatalytic tests

The photocatalytic activity of the prepared samples was evaluated by the degradation of Rhodamine B (RhB) in a beaker at room temperature. The standard procedure involved the addition of 20 mg of photocatalyst to 200 mL of an aqueous RhB solution (concentration: 7 mg L^−1^). The system was kept in the dark for 1 hour to allow adsorption/desorption equilibrium to be reached between the catalyst and the dye. The adsorbed dye was found to be negligible in relation to the overall reaction. The suspension was stirred and exposed to UV irradiation at an intensity of 50 W m^−2^ with a peak wavelength of 350 nm using an Osram ULTRA-Vitalux lamp (300 W). The lamp was turned on 30 minutes before the photocatalytic experiment to stabilize its output. A blank experiment, conducted without the catalyst, demonstrated that no photolytic degradation of RhB occurred. Analyses were performed using a quartz cuvette as the sample holder. At regular time intervals (1, 5, 10, 15, 20, 30, 40, 50, and 60 minutes), 3 mL of the solution was withdrawn, filtered through a 0.45 μm filter, and the absorbance at 554 nm was measured using a single-beam spectrophotometer (UV/Vis HachLange, DR 3900). The photocatalytic performance was quantified by calculating the photodegradation rate constant, *k* (min^−1^). The degradation of RhB in the presence of the catalyst is assumed to follow pseudo-first-order kinetics, as described by the eqn (5):5
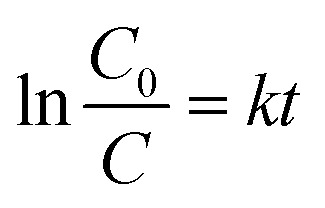


Based on the Lambert–Beer law, the absorbance is proportional to RhB concentration, so ln(*C*_0_/*C*) is calculated by measuring initial concentration (*C*_0_) and absorbance (*A*_0_) measured at a certain irradiation time *t* (*A*_*t*_). The value of *k* was assessed by plotting ln (*C*_0_/*C*) *versus* time (*t*). The conversion, calculated at *t* = 60 min, indicates the ratio between the amount of reagent consumed and the amount of reagent initially present in the reaction environment, and it was determined by formula (6):6
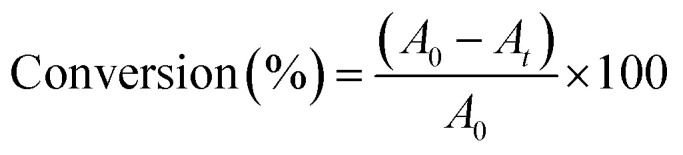


## 3. Results and discussion

### 3.1. Colloidal characterization of NeoC/TiO_2_ multicomponent suspensions

**Fig. 1:**
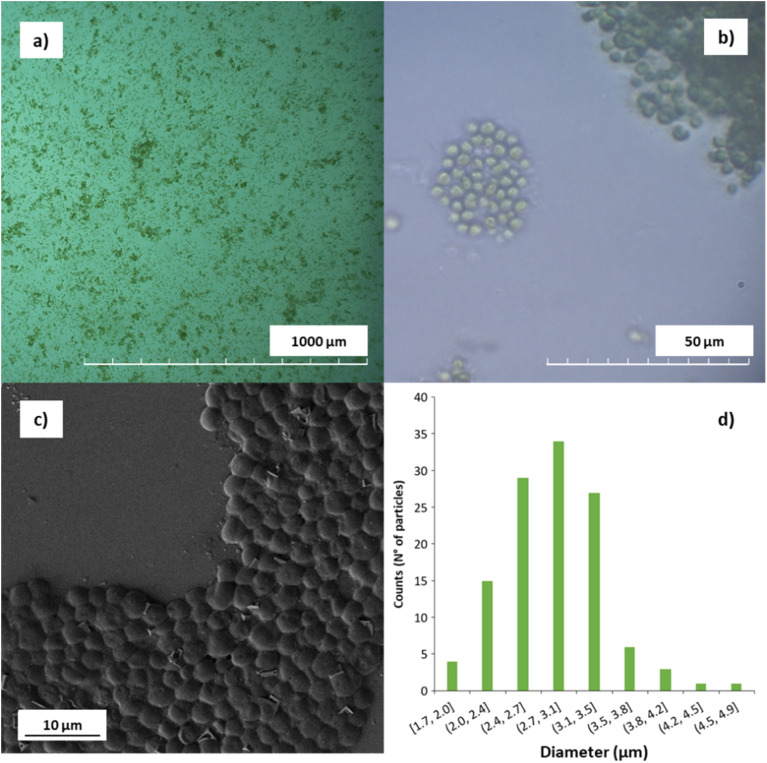
(a and b) Optical microscope image of *Neochloris oleoabundans* microalgae, (c) FESEM images of NoeC, and (d) dimensional analysis.

*Neochloris oleoabundans* (NeoC) is an oleaginous microalgal species that can be cultivated in fresh and saltwater.^19^ The optical microscope images of the microalgae (Fig. 1) highlighted the typical cells with a diameter of about 3 microns and spherical shape (Fig. 1a and b). FESEM images collected on the dried NeoC confirmed a mean diameter of 2.9 ± 0.5 μm and a spherical morphology of the cells (Fig. 1c and d). FT-IR spectra was collected on the *Neochloris oleoabundans* biomass to identify the principal functional groups (ESI, Fig. S1†).

**Fig. 2:**
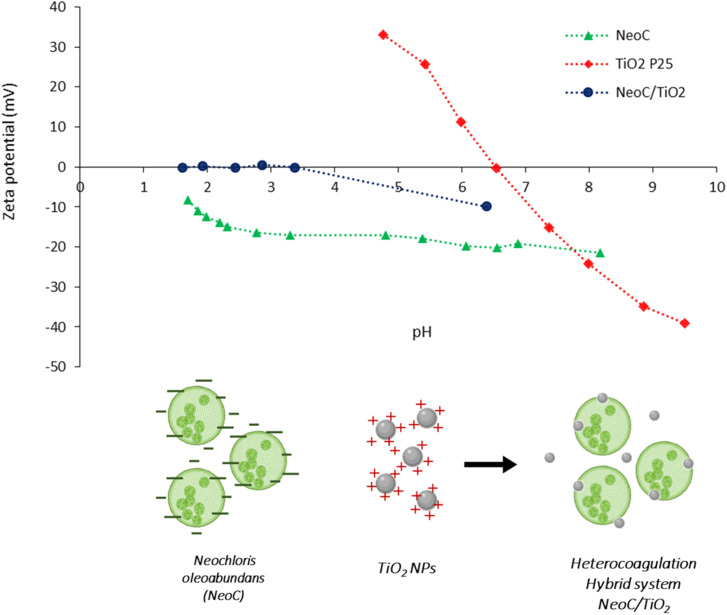
Z potential *vs.* pH titration curves of samples NeoC, TiO_2_ and hybrid sample NeoC/TiO_2_.

We coupled TiO_2_ NPs with NeoC biomass through a colloidal process driven by the heterocoagulation of the two components. Zeta Potential (ZP) titrations as a function of pH (Fig. 2) or the addition of NeoC (ESI, Fig. S2†) allowed us to investigate the colloidal behavior of the suspensions, which plays a key role in the study of the NeoC/TiO_2_ surface interaction.

NeoC exhibited a negative ZP of −21 mV at the natural pH of 8.5 and showed a negative ZP/pH profile across the entire explored pH range, with an isoelectric point (IEP) below 1.5 (Table 1 and Fig. 2) attributed to the negatively charged functional groups present on the microalgae surface. The negative surface charge of the microalgae cells contributes to promoting their electrostatic interaction with TiO_2_ NPs,^14^ which, in contrast, presented positive ZP values at acidic pHs and an IEP at pH 6.7 (Fig. 2). The hydrodynamic diameters highlighted a value of about 480 nm for TiO_2_ P25, in line with the agglomeration phenomena of the primary particles with real dimensions in the 20–25 nm range.^20^ Despite the low mass percentage of NeoC added to TiO_2_ in the NeoC/TiO_2_ sample, we observed strongly modified colloidal properties for the NeoC/TiO_2_ adducts, both in terms of ZP and hydrodynamic diameters, suggesting a significant electrostatic surface interaction between NeoC and TiO_2_. ZP exhibited an abrupt ZP switching to −10 mV and moved to a negative surface charge across almost the entire pH range with an IEP shift from 6.7 to 3.4 (Fig. 2). The ZP/pH curve of the hybrid sample (NeoC/TiO_2_) did not completely overlap the biomass curve, confirming that, at low biomass concentration, only a partial covering of the inorganic phase occurred. The presence of such a low biomass percentage also affected the hydrodynamic diameter, which increased to the micrometric range, reaching a dimension of about 4.5 μm, compatible with the NeoC cells.

**Table 1:** Zeta potential and hydrodynamic diameter of TiO_2_ P25, NeoC and NeoC/TiO_2_

Sample code	pH	ZP (mV)	*Φ*_DLS_ (nm)
TiO_2_ P25	5.8	+25.8 ± 0.2	482.8 ± 8.6
NeoC	8.5	−21.4 ± 2.2	13 340.7 ± 5020.3
NeoC/TiO_2_	6.4	−9.9 ± 0.4	4527.7 ± 947.3

### 3.2. Colloidal characterization of NeoC/TiO_2_–SiO_2_ multicomponent suspensions

**Fig. 3:**
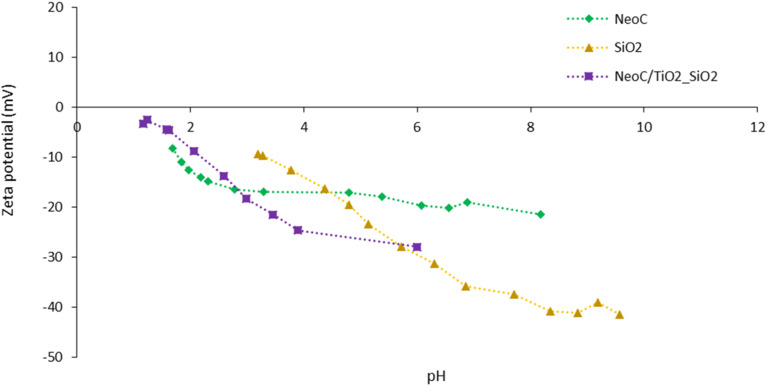
Zeta potential *vs.* pH titration curves of samples NeoC, SiO_2_ and NeoC/TiO_2_–SiO_2_.

After the first design of the NeoC/TiO_2_ sample, we developed a three-component sample based on TiO_2_, SiO_2_, and NeoC. The coupling of NeoC with a TiO_2_–SiO_2_ nanocomposite aimed to produce a hybrid composite with an increased photocatalytic activity. As reported in the literature, silica NPs are known to be a photocatalytic booster for titania NPs and can enhance their photocatalytic activity.^21^ Here, we observed a negative ZP for both components (TiO_2_–SiO_2_ and NeoC) along the whole pH range investigated (Fig. 3). In contrast to the NeoC/TiO_2_ coupling, which benefits from the attractive force between opposite surface charges, the interaction between the TiO_2_–SiO_2_ and NeoC phases is non-specific and most likely driven by the tendency of nanophases to reduce their surface free energy. The same amount of NeoC used to coat the TiO_2_ NPs surface, as derived by Fig. S2† (1.4 wt%) was used for preparing the NeoC/TiO_2_–SiO_2_ sample.

### 3.3. Morphology of the multicomponent hybrid granules

**Fig. 4:**
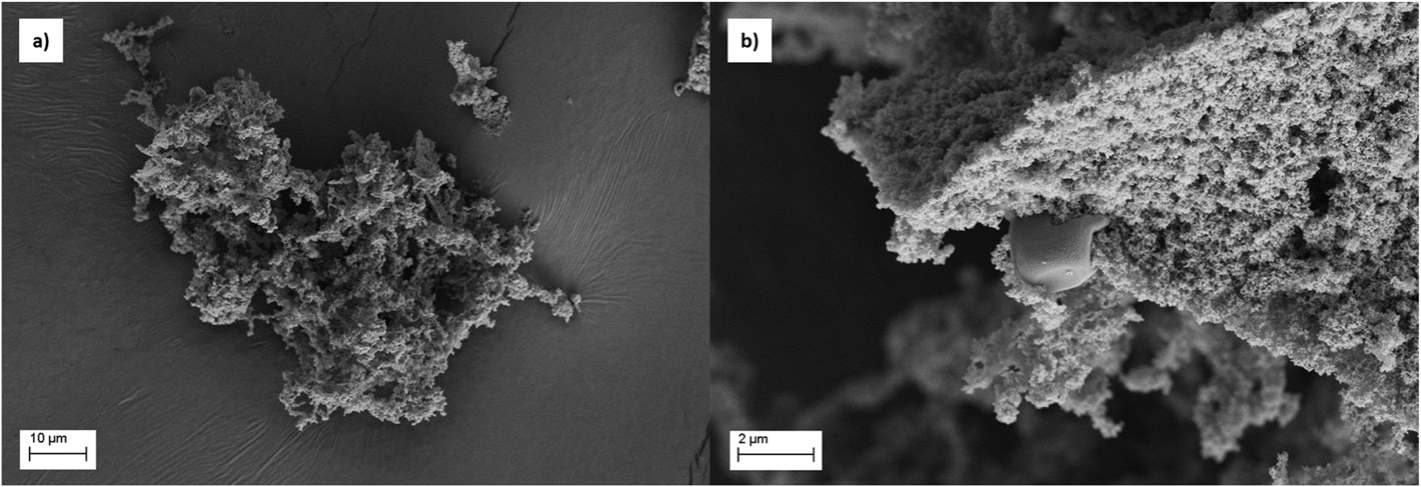
FESEM images of NeoC/TiO_2__SFD samples: (a) low magnification showing microaggregates; (b) high magnification revealing a nanostructured surface with embedded biomass integrated into the inorganic matrix.

**Fig. 5:**
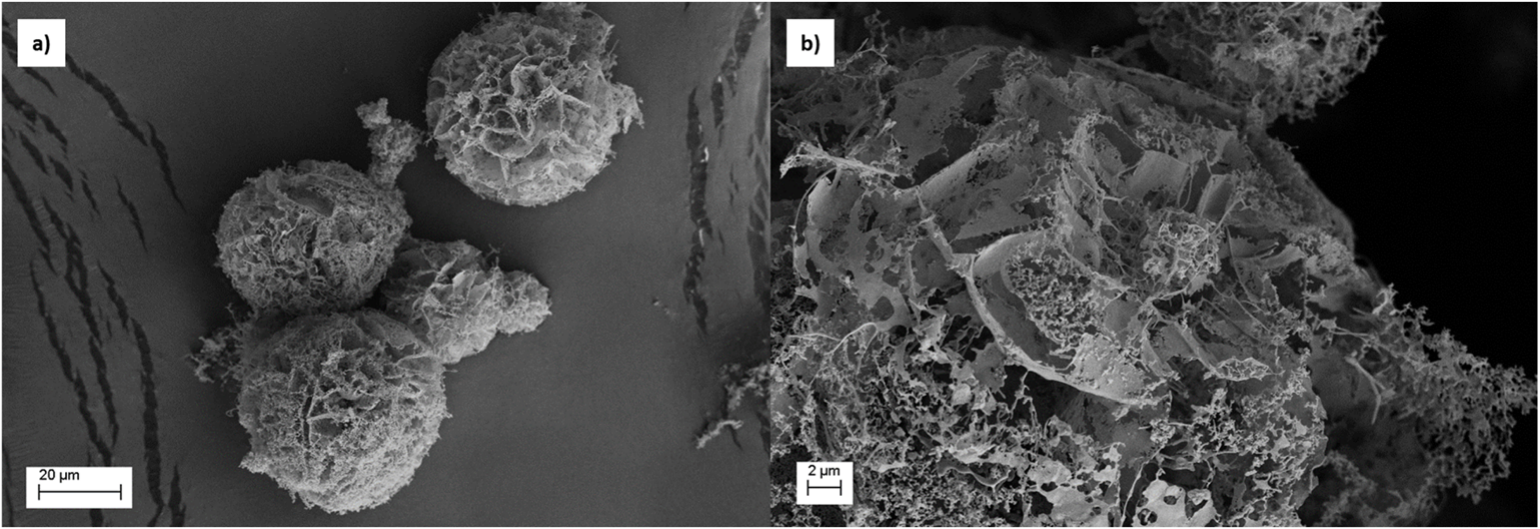
FESEM images of NeoC/TiO_2_–SiO_2__SFD samples: (a) low magnification showing spherical microgranules; (b) high magnification revealing a nanostructured surface with a highly porous architecture.

The prepared multicomponent suspensions, NeoC/TiO_2_ and NeoC/TiO_2_–SiO_2_, were granulated using a spray freeze drying process, an efficient method able to provide easy-to-handle micropowders (NeoC/TiO_2__SFD and NeoC/TiO_2_–SiO_2__SFD) preserving highly reactive porous nanostructures (Fig. 4 and 5) suitable to be applied as powder sorbent medium for water treatment.^22^ For NeoC/TiO_2__SFD (Fig. 4) we obtained TiO_2_-based nanostructures organized in micrometric granules embedding the biomass (Fig. 4b). As expected, the addition of SiO_2_ into NeoC/TiO_2_–SiO_2_ promoted the achievement of spherical and regular microgranules of about 20 μm with an increased mechanical resistance and surface area (Fig. 5 and Table 2).

**Table 2:** Specific surface area values measured on the prepared hybrid granules

Samples	Specific surface area (m^2^ g^−1^)
NeoC/TiO_2__SFD	67
NeoC/TiO_2_–SiO_2__SFD	385

### 3.4. Adsorption properties of the multicomponent hybrid granules

We evaluated the adsorption properties of the prepared hybrid granules in the removal of Cu^2+^ ions, selected as model heavy metal, due to the affinity of copper with *Neochloris oleoabundans*^18,19^ and other microalgae.^14,23^

We excluded any interferences with any heavy metals already adsorbed by the biomass by checking the NeoC's elemental composition [ESI, Table S1†].

**Fig. 6:**
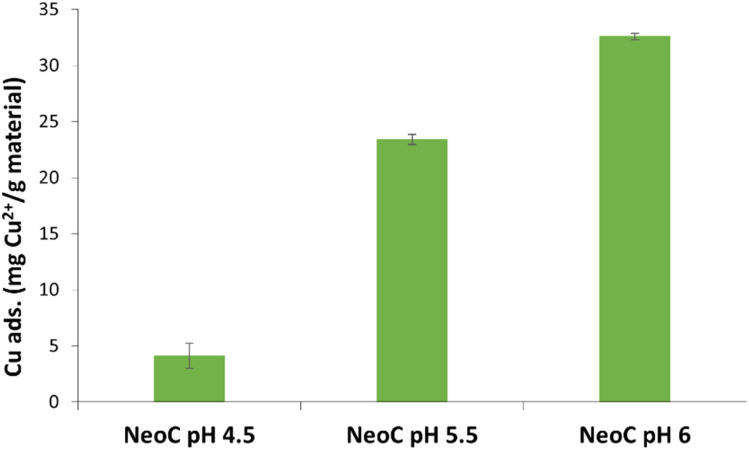
NeoC adsorption capacity measured for Cu^2+^ after 30 min of exposure at different pHs (4.5, 5.5 and 6).

We tested the prepared SFD hybrid granules by using a 10 mg per L copper solution. We selected pH 6 as the optimal working condition to prevent the precipitation of Cu(OH)_2_ and maximize the adsorption capacity of the microalgae.^18,19^ Solution pH is a crucial parameter in heavy metal adsorption phenomena, as it significantly impacts surface charge, dissociation of functional groups, and chemical reactions at the adsorbent surface.^24^ In this perspective, the effect of pH on the Cu^2+^ adsorption was evaluated at three values (4.5, 5.5, 6) and the results shown in Fig. 6 highlight pH 6 as the best adsorption condition for NeoC (approximately 30 mg Cu^2+^ per gram of material). This finding aligns with mechanisms reported in the literature: at more acidic pH, high concentrations of protons compete with metal ions for binding sites, whereas, as pH increases, the ionization of functional groups increases, providing more binding sites for Cu^2+^ ions.^19,25^ Furthermore, as shown in Table 1, all the materials analysed at this pH exhibit negative ZP values, suggesting a more favourable electrostatic interaction between the surface-exposed functional groups (negative charge) and the Cu^2+^ ions (positive charge).

Table 3 lists the adsorption data assessed for the components used to prepare the hybrid granules (NeoC, TiO_2_, SiO_2_, TiO_2_–SiO_2_) and the produced hybrid materials. As expected, the results confirmed that only NeoC has a relevant sorption capability for Cu^2+^ ions, adsorbing about 84 wt% of the initial Cu^2+^ content. The inorganic components, TiO_2_ and SiO_2_, evidenced low adsorption values of 0.45 and 0.64 mg g^−1^, respectively.

**Table 3:** Cu^2+^adsorption capacity measured for NeoC, inorganic and hybrid granules

Samples	Cu adsorption (mg_Cu_/g_materials_)	Cu adsorption (mg_Cu_/g_microalgae_)	Theoretical adsorption (mg_Cu_/g_materials_)
NeoC	32.59	32.59	—
TiO_2__SFD	0.45	—	—
SiO_2__SFD	0.43	—	—
TiO_2_–SiO_2__SFD	0.64	—	—
NeoC/TiO_2__SFD	4.41	276.95	1.65
NeoC/TiO_2_–SiO_2__SFD	4.17	247.34	1.83

The adsorption performance of the hybrid granules, NeoC/TiO_2__SFD and NeoC/TiO_2_–SiO_2__SFD, exhibited values around 4 mg g^−1^, indicating an adsorption capacity intermediate between NeoC and the inorganic counterpart. However, the adsorption performance of the hybrid granules containing 1.4 wt% of microalgae biomass was significantly higher than the theoretical expected values based on their weight composition (Table 3).

**Fig. 7:**
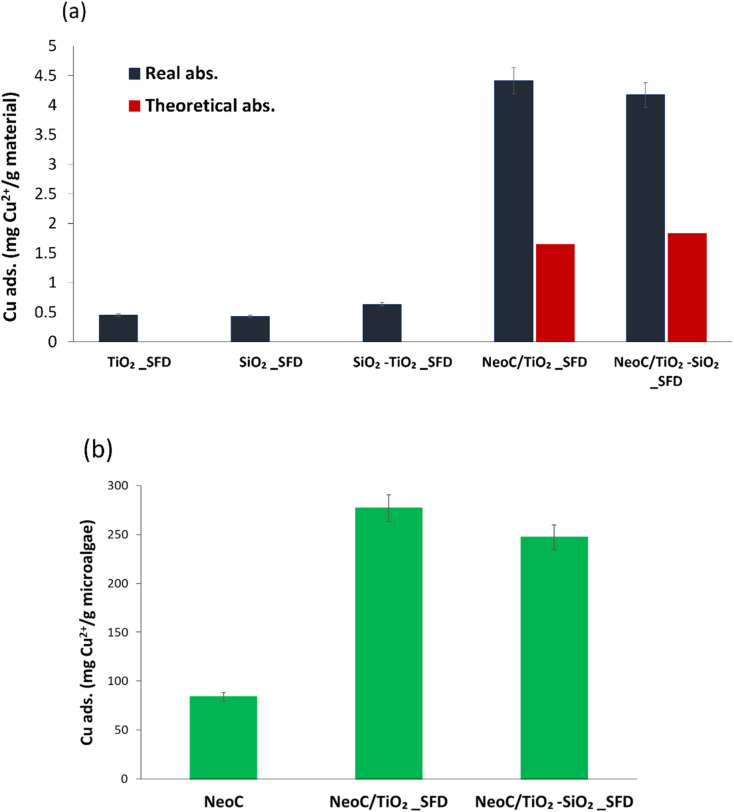
(a) Cu^2+^ adsorption measured for single components and hybrid granules, compared with the expected theoretical adsorption; (b) Cu^2+^ adsorption values normalized for gram of microalgae.

Fig. 7 shows that the adsorption values for the hybrid granules (blue bar) are markedly higher than the expected theoretical values (red bar). This behaviour can be attributed to a synergistic effect triggered by the coupling of the biomass with the inorganic nanophases and highlighted in Fig. 7a. Typical adsorption capacity values for bivalent metals by *Neochloris oleoabundans* are reported in the literature to range from 30 to 100 mg g^−1.^^19,26^ When NeoC interacts with the inorganic phase (samples NeoC/TiO_2_–SiO_2__SFD and NeoC/TiO_2__SFD) we observed a strong improvement in the biosorption capability in the range of 250–280 mg g^−1^, as already shown for *Chlorella vulgaris*.^14^

We hypothesize that the enhanced metal adsorption assessed for the microalgae is promoted by the action of the inorganic phase, which disperses the biomass more efficiently, thereby increasing the exposure of the functional groups in the cell wall responsible for heavy metal sorption. In NeoC/TiO_2_–SiO_2__SFD the high contribution of SiO_2_ to the specific surface area, which increased up to 380 m^2^ g^−1^ (Table 2), does not appear to significantly improve the adsorption performance, probably because the chemical interactions prevail over the physical contribution associated with the surface area.

To further highlight the synergistic adsorption effect triggered by coupling NeoC with the inorganic TiO_2_-based nanophases, we reported in Fig. 7b the Cu^2+^ adsorption value normalized per gram of microalgae. The histogram clearly shows for the hybrid granules a Cu^2+^ adsorption per gram of NeoC strongly increased if compared to the NeoC alone.

The effect of initial metal concentrations was evaluated under batch conditions at room temperature (pH = 6, sorbent dose = 2.5 g L^−1^, contact time 30 minutes). The Cu^2+^ concentration investigated ranged from 1 to 500 mg L^−1^, however, we only discussed the data collected in the 1–100 range, because CuCl_2_ precipitation occurring at high concentrations interferes with the adsorption assessment.

**Fig. 8:**
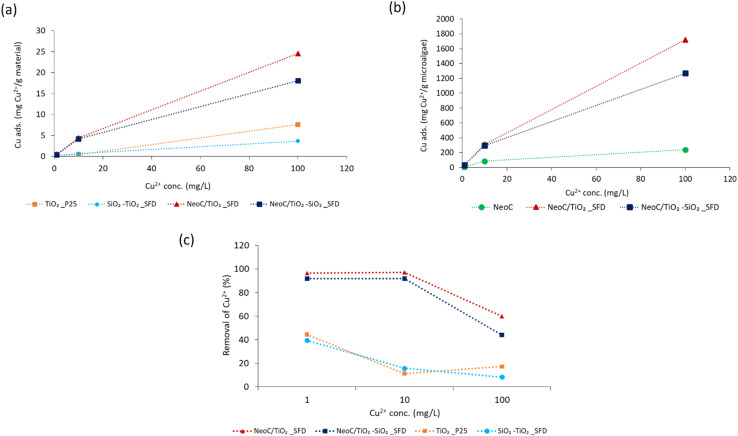
(a) Cu^2+^ adsorption capacity measured for hybrid and inorganic granules; (b) Cu^2+^ adsorption capacity normalized for gram of microalgae and (c) Cu^2+^ percentage removal measured for hybrid and inorganic granules at different Cu^2+^ concentrations.

Fig. 8a shows an increase in adsorption capacity as the concentration of copper ions (Cu^2+^) in the solution rises. This trend can be ascribed to a reduced mass transfer resistance between ions and bio-sorbents at higher metal ion concentrations.^25^ Consequently, the accessibility of the binding sites is enhanced, leading to a significant increase in adsorption capacity. The adsorption plateau, indicating that the binding sites become saturated, was not reached across the explored concentration range. The data from the hybrid granules confirm the advantage provided by the microalgae (Fig. 8a) as highlighted by the adsorption capacities of the hybrid materials (NeoC/TiO_2__SFD and NeoC/TiO_2_–SiO_2__SFD) significantly enhanced compared to the inorganic components alone (TiO_2_ and TiO_2_/SiO_2_). The synergistic effect detected on the hybrid granules was confirmed at the different Cu^2+^ concentrations. In fact, normalizing the adsorption on grams of biomass the values resulted markedly increased if compared to the NeoC alone (Fig. 8b).

Fig. 8c shows the same values as percentage adsorption: at 1 and 10 mg L^−1^ of Cu^2+^, the hybrid granules achieved complete removal, whereas the inorganic samples reached only 20–40% removal, reflecting their reduced adsorption capacity. At 100 mg L^−1^, the percentage removal for the hybrid samples decreased to 50–60%, because at lower initial concentration, a higher number of sites are available per mole of metal ions, but as moles of metal ion increase, binding sites become occupied leading to a decrease in the removal percentage.^27^

#### 3.4.1. Adsorption isotherms

Langmuir and Freundlich's isotherms were applied to the experimental data summarized in Fig. 8.

Table 4 shows the results obtained from the isotherm application with the corresponding correlation *R*^2^ values. In the Langmuir isotherm model, the values of *q*_max_, *b* and *R*^2^ were estimated from the linear plot between *C*_e_/*q*_e_ and *C*_e_, while for the Freundlich model the values of *n*, *K*_f_ and *R*^2^ were derived from the linear plots of log(*q*_e_) against log(*C*_e_). The Langmuir model provided high *R*^2^ values, however, for three samples, including NeoC and NeoC/TiO_2__SFD, it produced negative parameters, indicating that the model does not fit these sorbent materials.^28^ In contrast, the Freundlich model yielded a good fit for the multicomponent samples, including both the hybrid granules and the TiO_2_–SiO_2__SFD, suggesting multilayer sorption on heterogeneous surfaces. The *K*_f_ values for NeoC/TiO_2__SFD, NeoC/TiO_2_–SiO_2__SFD and TiO_2_–SiO_2__SFD were slightly lower than the assessed adsorption capacities, with values of 3.64, 2.06 and 0.20 mg g^−1^, respectively, but still the same order of magnitude. The 1/*n* parameter between 0 and 1 confirmed a favourable adsorption process.^29^ Although the NeoC/TiO_2_–SiO_2__SFD sample also yielded the same *R*^2^ value with the Langmuir model, the associated *q*_max_ did not correspond with the measured adsorption capacity, supporting the matching with the Freundlich isotherm. The Freundlich fitting was less precise for the NeoC biomass alone, associated with an *R*^2^ value of 0.7, but associated to a favourable adsorption (1/*n* = 0.66) and to a *K*_f_ of 16 mg g^−1^.

Langmuir and Freundlich parameters calculated on NeoC embedded into the hybrid samples NeoC/TiO_2__SFD and NeoC/TiO_2_–SiO_2__SFD

**Table:** 

Isotherm model	Sample code	*q*_max_ (mg g^−1^)	*b*	*R* ^2^
Langmuir	NeoC	−34.48	−0.21	0.89
	NeoC/TiO_2__SFD	−81.97	−0.11	0.99
	NeoC/TiO_2_–SiO_2__SFD	42.55	0.09	0.92
	TiO_2__SFD	1.01	0.38	0.72
	TiO_2_–SiO_2__SFD	−5.50	−0.64	0.97

**Table:** 

Isotherm model	Sample code	*K*_f_ (mg g^−1^)	1/*n*	*R* ^2^
Freundlich	NeoC	16.40	0.66	0.70
	NeoC/TiO_2__SFD	3.64	0.56	0.91
	NeoC/TiO_2_–SiO_2__SFD	2.06	0.58	0.92
	TiO_2__SFD	0.54	0.45	0.50
	TiO_2_–SiO_2__SFD	0.20	0.61	0.99

#### 3.4.2. Kinetic study

**Fig. 9:**
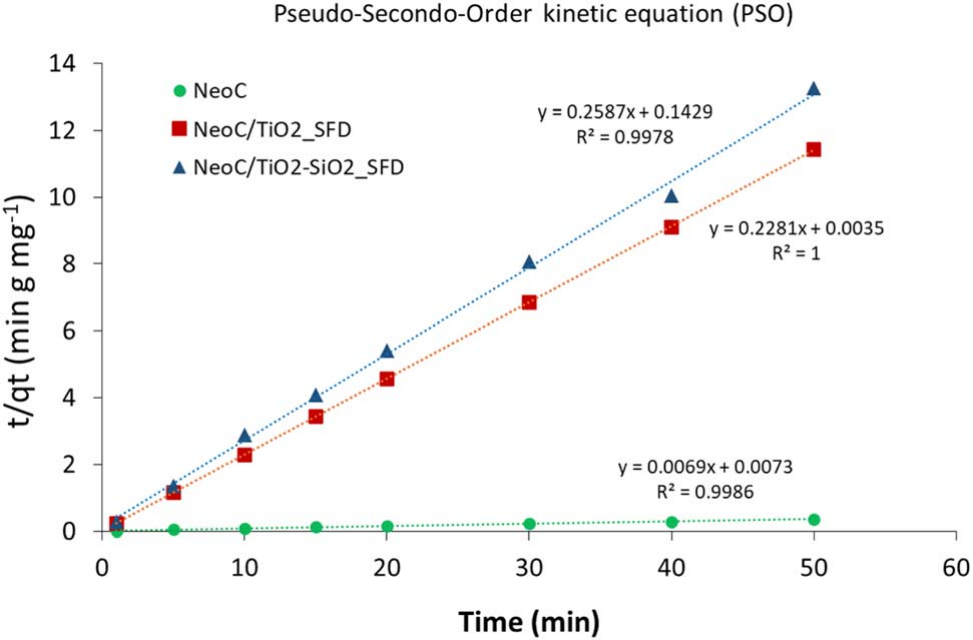
Cu^2+^ adsorption kinetics modelled in linear form according to pseudo-second-order kinetics (PSO).

The adsorption mechanism was analysed by two kinetic models: the pseudo-first-order (PFO) and the pseudo-second-order (PSO) kinetics. PFO kinetic model did not fit the data providing *R*^2^ in the 0.1–0.6 range, while the PSO model demonstrated an optimal correlation coefficient (*R*^2^) higher than 0.99 for all the measured samples best describing the adsorption kinetic of the prepared samples (Fig. 9). The PSO kinetic model is often associated with chemisorption processes where adsorption involves complex interactions, such as multilayer adsorption, where the limiting phase is the formation of chemical bonds between the adsorbate and adsorbent or the availability of active adsorption sites.^30,31^

The kinetic constant (*k*_2_) and the equilibrium adsorption (*q*_e_) values derived by applying the PSO model at the initial concentration of 10 mg L^−1^ are reported in Table 5. The *q*_e_ values are consistent with the measured data, and the very high kinetic constant observed for NeoC/TiO_2__SFD suggests that for this sample, the adsorption rate is particularly fast during the early stages of the process. This behaviour is typical of systems where chemisorption occurs, indicating that the adsorption sites are highly reactive and readily available.^32^ The introduction of SiO_2_ reduced both the adsorption capacity and the adsorption rate.

**Table 5:** PSO Kinetic model applied to NeoC, and hybrid samples NeoC/TiO_2__SFD and NeoC/TiO_2_–SiO_2__SFD

Kinetic model	Sample	*q*_e_ (mg g^−1^)	*k*_2_ (g min^−1^ mg^−1^)	*R* ^2^
Pseudo 2nd Order	NeoC	14.39	0.0065	0.99
	NeoC/TiO_2__SFD	4.38	14.86	1
	NeoC/TiO_2_–SiO_2__SFD	3.82	0.46	0.99
	TiO_2__SFD	0.33	−0.62	0.95
	TiO_2_–SiO_2__SFD	1.03	1.31	0.99

**Fig. 10:**
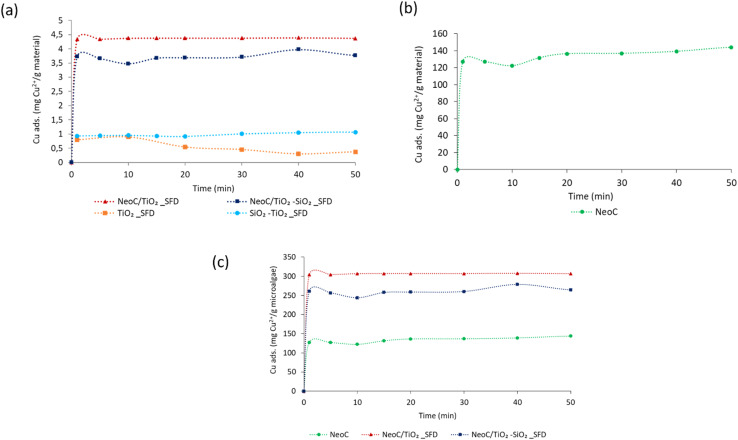
Cu^2+^ absorption capacity time-dependent measured for all the samples: (a) for gram of material measured for hybrid and inorganic granules, (b) for gram of the biomass alone, (c) normalized for gram of microalgae measured for hybrid and biomass alone.

As discussed earlier, both hybrid samples exhibited an adsorption capacity higher than expected based on their composition (Fig. 10). This increase is attributed to an improved dispersion of the biomass, which promotes greater exposure of the key functional groups. The plateau was reached within the first 5 minutes, with no signs of destabilization, as the adsorption capacity remained constant throughout the test duration (50 minutes). This indicates good adsorption performance for all samples over time, without any relevant desorption phenomenon occurring within 50 minutes.

### 3.5. Photocatalytic tests of the multicomponent hybrid granules

**Fig. 11:**
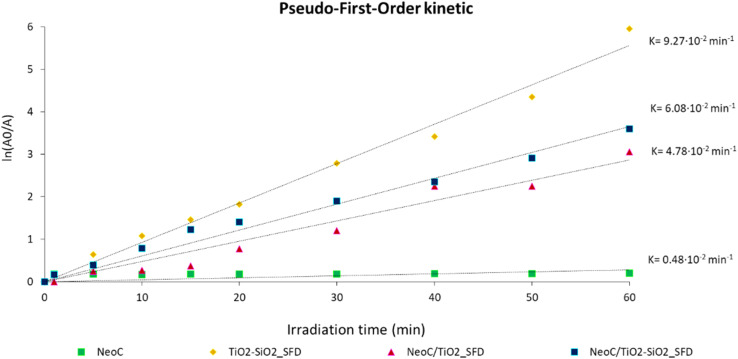
Photocatalytic performances collected on RhB photodegradation for NeoC, TiO_2_–SiO_2_, and hybrid granules NeoC/TiO_2_ and NeoC/TiO_2_–SiO_2_.

We tested the photocatalytic activity of the SFD hybrid granules to determine whether the microalgae affect the TiO_2_ performance. We investigated the performance under UV irradiation (Fig. 11 and Table 6) by monitoring the photodegradation of Rhodamine B (RhB), a model molecule that is easily detectable using a spectrophotometric method. As expected, the data collected on the single components evidenced no photocatalytic activity for the microalgae alone, NeoC, while confirmed the excellent performance for the SFD granules of the inorganic counterparts. Both TiO_2__P25 and TiO_2_–SiO_2_ showed a pseudo-first-order kinetic profile,^33^ kinetic constants of 8.70 and 9.27 × 10^−2^ min^−1^, respectively, associated with and a total conversion reached within 60 min of irradiation. Data showed that the presence of NeoC in the hybrid granules induced lower kinetic constants (*k*) (Table 6), but without suppressing the photocatalytic activity that was merely delayed, as confirmed by the almost complete conversions reached within 60 minutes. The lowering of the kinetic constant was probably due to the interaction of the biomass with TiO_2_ at the surface, shielding the inorganic photocatalyst activation and thus preventing an optimal UV irradiation.^34^ The addition of silica in NeoC/TiO_2_–SiO_2__SFD counteracted the detrimental effect induced by the biomass, improving the photocatalytic performance of TiO_2_ (ref. 33) and the resulting kinetic constant value of the hybrid sample (Table 6 and Fig. 11). The presence of silica enhances the photocatalytic performance of this hybrid material, making it comparable to similar TiO_2_/SiO_2_ structures.^17,35^ Simultaneously, it imparts adsorption functionality, paving the way for promising applications in wastewater treatment. Specifically, SiO_2_ NPs enhances the photocatalytic performance of TiO_2_ by increasing the specific surface area, thereby improving the availability of reactive sites. Additionally, the presence of SiO_2_ NPs suppresses H_2_O_2_ formation at the particle interface, favoring the generation of reactive oxygen species such as superoxide (˙O_2_^−^) and hydroxyl (˙OH) radicals, which collectively contribute to enhanced photocatalytic activity.^36^

**Table 6:** Photocatalytic data collected on the prepared hybrid materials after spray freeze drying. Kinetic constants are calculated on 60 min of reaction

Sample	*k* × 10^−2^ (min^−1^)	Conversion 60 min (%)
TiO_2__P25_SFD	8.70	99.0
TiO_2_–SiO_2__SFD	9.27	100
NeoC	0.48	18.6
NeoC/TiO_2__SFD	4.78	95.3
NeoC/TiO_2_–SiO_2__SFD	6.08	97.3

## 4. Conclusions

We developed hybrid granules by coupling *Neochloris oleoabundans* biomass with TiO_2_ and TiO_2_–SiO_2_ inorganic nanophases, resulting in novel sorbent/photocatalytic materials for water treatment applications. The combination of *Neochloris oleoabundans* biomass with the inorganic nanophases was performed at the colloidal stage to promote surface interactions. The mixture was then dried using a spray-freeze drying technique, which produced nanostructured porous granules.

The hybrid granules exhibited higher Cu^2+^ adsorption than expected based on their compositional percentage. This enhancement is likely due to a synergistic effect occurring when the microalgae biomass is embedded in the inorganic phase. The increased adsorption can be attributed to better dispersion of the biomass, facilitated by the inorganic phase acting as a support. This improves the interaction between the biomass and metal ions, significantly enhancing the adsorption capacity of the microalgae from about 30 mg g^−1^ to 250 mg g^−1^. Despite the presence of SiO_2_, which drastically increased the overall specific surface area of the inorganic component, the NeoC/TiO_2_ sample exhibited the highest ion adsorption capacity. This suggests that chemical interaction mechanisms, modeled by Freundlich isotherm and pseudo-second-order kinetic, dominate over the physical contribution of the high surface area.

On the other hand, from the photocatalytic perspective, the addition of SiO_2_ boosted the photocatalytic activity, which was slightly hindered by the presence of the microalgae. Specifically, the interaction between the biomass and TiO_2_ was detrimental to the photocatalytic performance, leading to a reduced photodegradation rate, although the process still achieved nearly complete conversion within 1 hour. This reduction was mitigated by incorporating silica nanoparticles, which helped restore the photocatalytic performance, providing effective sorbent and photocatalytic materials.

The use of non-living microalgae to integrate two functionalities into a synergistic system opens up promising new opportunities for microalgae in water treatment applications, significantly increasing the value of the proposed technology from a sustainability perspective.

The potential for integrating microalgae biomass with inorganic nanophases also paves the way for their application in other emerging fields, such as regenerative agriculture and nutraceutical supplements. In these contexts, the inert inorganic nanophase can preserve, encapsulate, and gradually release the microalgal nutrients.

## Conflicts of interest

The authors declare that they have no known competing financial interests or personal relationships that could have appeared to influence the work reported in this paper.

## Supplementary Material

NA-007-D5NA00236B-s001

NA-007-D5NA00236B-s002

NA-007-D5NA00236B-s003

NA-007-D5NA00236B-s004

## Data Availability

Data will be made available on request.
