# Supplementary figures and images for: Hybrid materials for wastewater treatment: synergistic coupling of Neochloris oleoabundans and TiO2 nanoparticles
Source: Nanoscale Adv. 2025 May 12;7(12):3803–16. doi: 10.1039/d5na00236b (PMC12086446; doi:10.1039/d5na00236b)

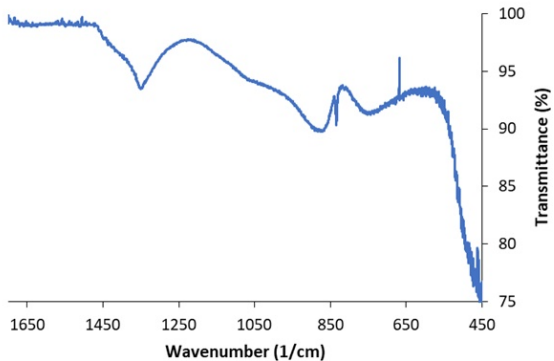

Supplement: NA-007-D5NA00236B-s002 [file NA-007-D5NA00236B-s002.pdf]

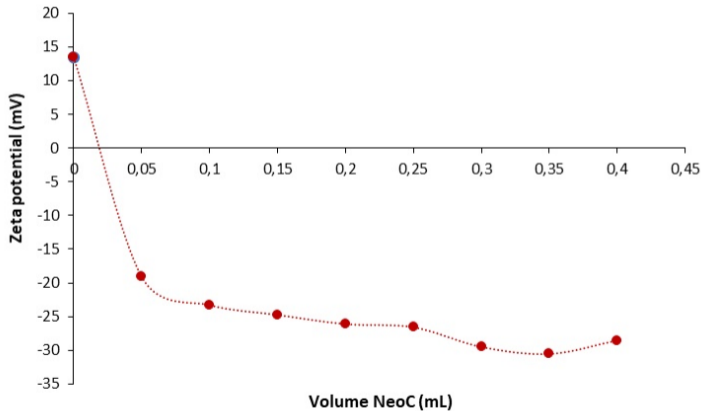

Supplement: NA-007-D5NA00236B-s003 [file NA-007-D5NA00236B-s003.pdf]
