# Supplementary material for: Hybrid materials for wastewater treatment: synergistic coupling of Neochloris oleoabundans and TiO2 nanoparticles
Source: Nanoscale Adv. 2025 May 12;7(12):3803–16. doi: 10.1039/d5na00236b (PMC12086446; doi:10.1039/d5na00236b)
Supplement: NA-007-D5NA00236B-s004 [file NA-007-D5NA00236B-s004.pdf]

Supplementary Information (SI) for Nanoscale Advances.
This journal is © The Royal Society of Chemistry 2025

Electronic Supplementary Information (ESI)

Hybrid Materials for Wastewater Treatment: Synergistic Coupling of *Neochloris oleoabundans* and Titania Nanoparticles

Ilaria Zanoni[1,#], Sara Amadori[1,2#], Andrea Brigliadori[1], Anna Luisa Costa[1], Simona Ortelli[1], Pierluigi Giacò[3], Costanza Baldisserotto[3], Simonetta Pancaldi[3], Magda Blosi[1*]

[1] CNR-ISSMC, National Research Council of Italy-Institute of Science, Technology and Sustainability for Ceramics, Faenza, Italy

[2] Department of Chemical Science, Life and Environmental Sustainability, Parma University, Parma, Italy

[3] Department of Environmental and Prevention Sciences, University of Ferrara, 44121, Ferrara, Italy

*Correspondence: magda.blosi@issmc.cnr.it; #: These authors share first authorship

1. FT-IR analysis of *Neochloris oleoabundans*

FTIR spectra of NoeC microalgae are shown in Figure S1. NeoC spectra presented sharp peaks at 834 and 1346 cm^{-1} that were associated respectively with C-N and S=O bonds, typical functional groups of nucleic acids, phospholipids, phosphoprotein, and phosphosaccharides [1,2].

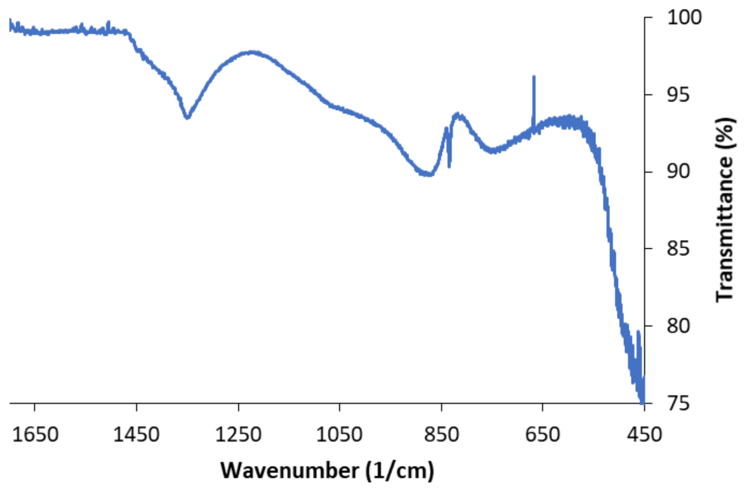

Figure S1*: FTIR spectrum of Neochloris oleoabundans*

2. Zete Potential titration curve of NeoC added with TiO_2 suspension

To explore the NeoC/TiO_2 surface interaction and identify the amount of biomass needed to maximize it, we titrated the TiO_2 suspension (0.1 g L^{-1}) with the NeoC one (0.22 g L^{-1}). When the first 50 µL of NeoC were added, a sudden decrease in the ZP value was observed, as shown by the ZP/pH curve (Figure S2). At higher NeoC concentrations, the zeta potential stabilizes between -20 and 30 mV, reaching a plateau consistent with the complete coating of the TiO_2 surface. In designing the NeoC/TiO_2 system, we selected the minimum amount of NeoC necessary to significantly modify the TiO_2 surface, corresponding to 60 µL (1.4% wt.).

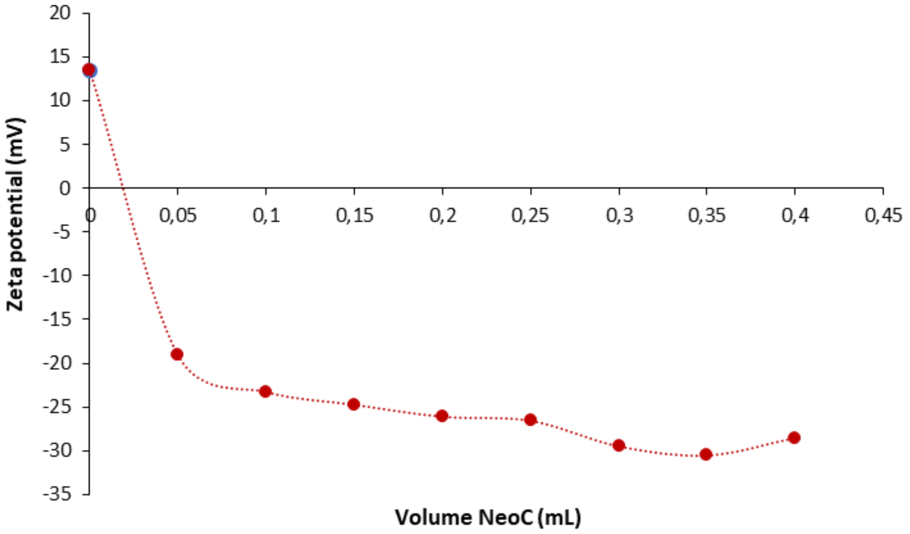

Figure S2: *Titration curve measured for TiO_2 NPs (0.1 g L^{-1}) added with NeoC (0.22 g L^{-1}).*

3. ICP analysis of *Neochloris oleoabundans* biomass

The NeoC microalgae's elemental composition was evaluated to assess the presence of any heavy metals in the biomass and exclude the presence of copper, used as probe ion to measure the adsorption capacity of the microalgae. As reported in Table S1, the main component is Na; while Ca, K, and Mg were detected as trace elements at low percentages (< 5% wt).

Table S1: Elemental composition of NeoC expressed as weight percentage (%wt).

Samples	Ca (%wt)	K (%wt)	Mg (%wt)	Na (%wt)
**NeoC**	4.5 ± 0.1	1.6 ± 0.1	1.5 ± 0.1	25.0 ± 0.2

References

1. Gu, S., Boase, E. M. & Lan, C. Q. Enhanced Pb(II) removal by green alga Neochloris oleoabundans cultivated in high dissolved inorganic carbon cultures. *Chem. Eng. J.* **416**, 128983 (2021).
2. Gu, S. & Lan, C. Q. Biosorption of heavy metal ions by green alga Neochloris oleoabundans: Effects of metal ion properties and cell wall structure. *J. Hazard. Mater.* **418**, 126336 (2021).
